# Glycolysis Reprogramming in Idiopathic Pulmonary Fibrosis: Unveiling the Mystery of Lactate in the Lung

**DOI:** 10.3390/ijms25010315

**Published:** 2023-12-25

**Authors:** Peishuo Yan, Jingyi Liu, Zhenwei Li, Jiawei Wang, Zhao Zhu, Lan Wang, Guoying Yu

**Affiliations:** State Key Laboratory of Cell Differentiation and Regulation, Henan Center for Outstanding Overseas Scientists of Organ Fibrosis, Pingyuan Laboratory, College of Life Science, Henan Normal University, Xinxiang 453007, China; yanpeishuo@stu.htu.edu.cn (P.Y.); 2204183002@stu.htu.edu.cn (J.L.); 2204183024@stu.htu.edu.cn (Z.L.); wangjiawei@stu.htu.edu.cn (J.W.); zhuzhao@stu.htu.edu.cn (Z.Z.)

**Keywords:** IPF, metabolism, glycolysis reprogramming, lactate, pathogenesis

## Abstract

Idiopathic pulmonary fibrosis (IPF) is a chronic and progressive lung disease characterized by excessive deposition of fibrotic connective tissue in the lungs. Emerging evidence suggests that metabolic alterations, particularly glycolysis reprogramming, play a crucial role in the pathogenesis of IPF. Lactate, once considered a metabolic waste product, is now recognized as a signaling molecule involved in various cellular processes. In the context of IPF, lactate has been shown to promote fibroblast activation, myofibroblast differentiation, and extracellular matrix remodeling. Furthermore, lactate can modulate immune responses and contribute to the pro-inflammatory microenvironment observed in IPF. In addition, lactate has been implicated in the crosstalk between different cell types involved in IPF; it can influence cell–cell communication, cytokine production, and the activation of profibrotic signaling pathways. This review aims to summarize the current research progress on the role of glycolytic reprogramming and lactate in IPF and its potential implications to clarify the role of lactate in IPF and to provide a reference and direction for future research. In conclusion, elucidating the intricate interplay between lactate metabolism and fibrotic processes may lead to the development of innovative therapeutic strategies for IPF.

## 1. Introduction

Idiopathic pulmonary fibrosis (IPF) is a chronic, progressive, and highly fatal lung disease characterized by progressive lung scarring and histological features of usual interstitial pneumonia (UIP) [1]. The main features of IPF include apoptosis of alveolar epithelium, accumulation of fibroblastic foci, and excessive deposition of extracellular matrix proteins, leading to the destruction of alveolar structure and irreversible loss of function [2,3]. Although this disease is considered rare, its incidence is similar to that of gastric cancer, brain cancer, and testicular cancer [4,5]. Currently, there are only two FDA-approved drugs for treating IPF: nintedanib and pirfenidone [6]. However, these two drugs cannot cure the disease but only moderately slow the decline in lung function. Therefore, elucidating its pathogenesis and being able to diagnose and treat it effectively in a timely manner are the biggest challenges faced by the clinical and research fields.

The pathogenesis of IPF is highly complex and is associated with the mechanisms and progression of pulmonary fibrosis caused by inhaled particles. Additionally, the incidence of idiopathic pulmonary fibrosis is correlated with a history of smoking in the majority of patients. Various other environmental exposures are also associated with IPF, including exposure to metals and wood dust, agriculture and farming, viruses, and silica [7,8,9,10]. Increasing evidence suggests that genetic susceptibility plays a role in the development of idiopathic pulmonary fibrosis, and studies on familial interstitial pneumonia have identified rare genetic variations [11]. Historically, IPF was considered a chronic inflammatory disease that eventually progressed to fibrosis of the lungs. However, subsequent research has revealed that anti-inflammatory treatment alone does not improve IPF, and the combination of immunosuppressive therapy with prednisolone and azathioprine significantly improves patient mortality rates [12]. Currently, IPF is generally considered to be the result of multiple interacting genetic and environmental risk factors, and the dysregulation of Type II alveolar epithelial cells (AEC2s) is believed to be the core mechanism underlying the pathogenesis of IPF.

There is an increasing recognition of the significant role that metabolic dysregulation plays in the pathogenesis of many pulmonary diseases, such as pulmonary fibrosis, pulmonary arterial hypertension, and chronic obstructive pulmonary disease [13,14,15,16]. One of the most prominent features of IPF is metabolic dysregulation within the lungs, characterized by mitochondrial dysfunction and metabolic reprogramming in different IPF lung cells (alveolar epithelial cells, fibroblasts, and macrophages), which increases susceptibility to activation of fibrotic responses. There is evidence indicating metabolic dysregulation of glucose, lipids, hormones, and other metabolites in the lungs of IPF patients [17,18,19,20,21]. The metabolic abnormalities within the alveoli lead to significant fluctuations in the levels of various metabolic products, severely impacting the homeostasis of the pulmonary microenvironment [22,23,24,25]. As an inevitable byproduct of glycolysis, lactate is one of the most abundant cellular metabolites in the lung tissue of IPF patients, playing a significant role in the pulmonary microenvironment. Although previous studies have indicated the involvement of lactate in the progression of IPF, its actions in this unique pulmonary microenvironment are complex and diverse. This review aims to explore the relationship between glycolytic reprogramming and IPF, and comprehensively analyze the research progress and potential significance of lactate in IPF.

## 2. Aerobic Glycolysis

After the uptake of carbohydrate by mammalian cells, it undergoes digestion and processing by various glucosidases, resulting in the production of monosaccharides, primarily glucose. These monosaccharides are then transported to various tissues in the body. Upon cellular uptake, glucose undergoes multiple enzymatic reactions in the cytoplasm, where it is metabolized into pyruvate. In tissues with high mitochondrial abundance, cytoplasmic pyruvate is transported into the mitochondrial matrix, where it is converted to acetyl-CoA by the pyruvate dehydrogenase complex. Acetyl-CoA, along with oxaloacetate, enters the tricarboxylic acid cycle, generating a significant amount of ATP. Under certain conditions, such as in mitochondria-deficient red blood cells or cells experiencing ischemic or hypoxic conditions, pyruvate is reduced to lactate in the cytoplasm, accompanied by the production of a small amount of ATP, and this process is known as anaerobic glycolysis. The general consensus is that anaerobic glycolysis occurs only in hypoxic tissues. However, in the twentieth century, Otto Warburg first observed that tumors consume more glucose than surrounding normal tissues, and even under normoxic conditions, glucose in cancer cells is largely converted to lactate instead of being utilized for oxidative phosphorylation (OXPHOS). This phenomenon is known as aerobic glycolysis or the “Warburg effect” [26].

Warburg’s hypothesis that aerobic glycolysis in cancer cells is a consequence of irreversible mitochondrial damage has been largely refuted based on evidence that cancer cells possess intact mitochondrial function and can generate ATP from both glycolysis and OXPHOS [27]. The occurrence of aerobic glycolysis can be attributed to the increased metabolic demand for ATP in proliferating cells, such as tumor cells, therefore, glycolysis is highly active in proliferating cells [28]. There is increasing evidence that stromal cells, particularly cancer-associated fibroblasts (CAFs) in surrounding tumor tissue exhibit high rates of aerobic glycolysis, leading to lactate secretion to adjacent cancer cells, thereby promoting cancer cell growth. This phenomenon is also known as the “reverse Warburg effect”, and the loss of Caveolin-1 protein in CAFs is believed to be the basis of the “reverse Warburg effect” [29]. Similarly, the reverse Warburg effect is also observed in pulmonary fibrosis, where studies have shown increased glycolysis in fibrotic lung fibroblasts (FLFs) isolated from fibrotic lung explants of IPF patients or various mouse models of pulmonary fibrosis. The caveolin-1 scaffolding domain peptide CSP7 can restore *p53* and miR-34a expression, thereby inhibiting abnormal glycolysis in pulmonary fibrosis by reducing the elevation of glycolytic enzymes [30].

## 3. Glycolysis Reprogramming in Lung Fibrosis

The lung is often overlooked as a metabolically active organ, but biochemical studies have long shown that glucose utilization in the lung exceeds that of many other organs, including the heart, kidneys, and brain. While many pulmonary diseases are now associated with cellular metabolic abnormalities, it is only recently that the concept of metabolic dysfunction has been recognized as a driving factor in the pathology of IPF. Studies have shown that IPF patients exhibit higher glycolytic activity in fibrotic areas, and glycolytic capacity has been identified as a predictive indicator of lung function decline and higher mortality rates [31]. Glucose uptake is the first rate-limiting step in glycolysis, and glucose transporters (GLUTs) are the most abundant and widely distributed glucose transporters in mammalian cells. Dysregulation of GLUT expression is associated with various pathological conditions. In various malignancies, aberrantly high expression of GLUTs, particularly GLUT1, can increase glucose uptake and metabolism, promoting tumor cell growth and leading to decreased survival rates [32,33,34]. Furthermore, early studies have reported that TGF-β can induce the level of GLUT1 in fibroblasts and mesenchymal cells [35,36]. Researchers found enhanced expression of GLUT1 in the lungs of patients with IPF and in a bleomycin-induced pulmonary fibrosis mouse model. Additionally, TGF-β induction increased mRNA and protein levels of GLUT1 in mouse fibroblast cell lines and primary cells, and enhanced glucose uptake [37]. IPF is often considered an age-related disease, in which aging and mitochondrial dysfunction can promote fibrosis. Cho found that a GLUT1-dependent glycolytic phenotype in the lungs of aged mice was significantly higher compared with young mice, which may be associated with increased sensitivity to bleomycin in aged mice [38]. Genetic and pharmacological inhibition of GLUT1 suppressed glycolytic activation in primary mouse lung fibroblasts and myofibroblast activation, and significantly inhibited bleomycin-induced pulmonary fibrosis in vivo. They further discovered that GLUT1-dependent glycolysis mediates acute exacerbation of pulmonary fibrosis caused by Streptococcus infection. Inhibiting GLUT1-dependent glycolysis limits the activation of macrophage AIM2 (Absent in Melanoma 2) inflammasomes, and specifically eliminating GLUT1 in bone marrow cells improves exacerbated fibrosis due to Streptococcus pneumoniae infection. These results suggest that inhibiting GLUT1-mediated glycolysis in macrophages is a viable therapeutic approach for treating IPF [39].

**Figure 1:**
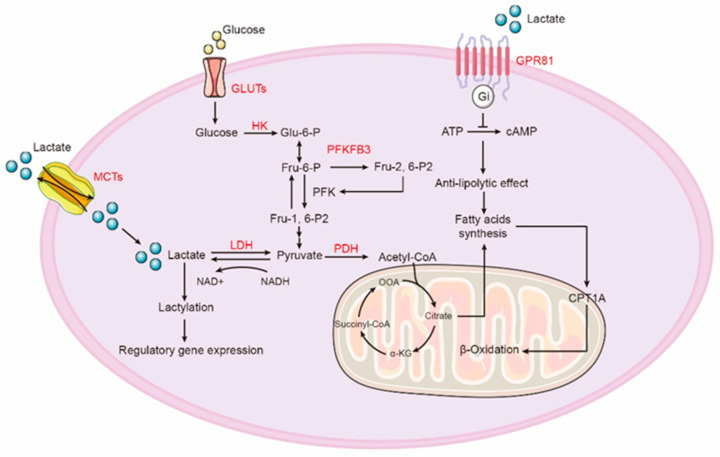
Cellular glycolysis and lactate metabolism play important roles in cellular energy metabolism. Cells take up glucose and generate pyruvate, which is then catalyzed by lactate dehydrogenase to produce lactate. Cells can export or uptake lactate through monocarboxylate transporters (MCTs), and the lactate taken up by cells is oxidized to pyruvate, entering the TCA cycle. Additionally, the lactate receptor GPR81 on the cell membrane can receive lactate signals and inhibit intracellular lipolysis by suppressing cAMP production.

After transported into cells by GLUTs, glucose undergoes glycolysis under the action of various enzymes, ensuring the occurrence of glycolysis (Figure 1). The abnormal expression of these key glycolytic enzymes in pulmonary fibrosis may regulate the progression of fibrosis. After comprehensive analysis of the gene expression profile and major metabolic programs of alveolar macrophages in experimental pulmonary fibrosis mice, researchers found that alveolar macrophages in fibrotic lungs induced by bleomycin and active TGF-β1 exhibited predominant pro-fibrotic M2-like characteristics. Furthermore, these fibrotic alveolar macrophages were often accompanied by significantly enhanced glycolysis and upregulation of various key glycolytic intermediates [40]. Similarly, in macrophages from silica-induced rat lungs, there is abnormal elevation of key glycolytic enzymes HK2, PKM1, and LDHA. Inhibition of LDHA can reduce silica-induced glycolysis and enhance macrophage activation, thereby inhibiting the progression of silicosis in rats [41]. As a typical pro-fibrotic cytokine, TGF-β1 can promote glucose consumption in fibroblasts. It has been shown to induce an increase in lactate levels and enhance the expression levels of glycolytic enzymes, including HK1, phosphofructokinase (PFKM), PKM1, and pyruvate dehydrogenase kinase isoform 1 (PDK5) [42]. Moreover, the glycolysis inhibitor 2-deoxy-D-glucose (2-DG) has been shown to inhibit the production of fibrotic markers and cell proliferation induced by TGF-β1, indicating that aberrant glycolysis contributes to the activation of fibroblasts [43].

Hexokinases (HKs) catalyze the first obligatory step in glucose metabolism, generating glucose-6-phosphate as the first intermediate for major biological processes involving glucose. Yin et al. found that HK2 levels are elevated in lung fibroblasts of patients with IPF. TGF-β induces HK2 accumulation and stimulates glycolysis in mouse and human lung fibroblasts. Treatment with the HK2 inhibitor lonidamine reduced the expression of pro-fibrotic genes and stabilized lung function in a mouse model of bleomycin-induced pulmonary fibrosis [44]. YAP/TAZ have been identified as sensors and mediators of extracellular matrix stiffness or other mechanical stresses in cells, and they can enhance fibrosis development by activating the expression of relevant genes through their nuclear translocation [45,46,47]. This process plays a crucial regulatory role in many biological processes, including cell migration, proliferation, tissue development, and physiological function regulation. Integrins, YAP/TAZ, TRPV4, and other key effectors are responsive to mechanical stimuli. Integrins can transmit mechanical and biochemical signals into cells, promoting cell proliferation, differentiation, migration, and invasion. Cells adjust their tension state through focal adhesions (FA) mediated by integrins to respond to ECM rigidity, triggering downstream cascades. During this process, actomyosin tension increases and reorganizes the entire cellular cytoskeleton, facilitating the nuclear translocation of YAP/TAZ transcriptional co-activators to promote the transcription of downstream genes involved in cell proliferation, collagen synthesis, and cell differentiation [48]. In the progression of pulmonary fibrosis, the deposition of extracellular matrix leads to an increase in ECM stiffness, activating integrins, and subsequently activating YAP/TAZ to promote the activation of fibroblasts and epithelial cells in lung tissue [49]. Additionally, integrins can influence cellular glycolysis processes by regulating various signaling pathways such as PI3K/Akt, MAPK/ERK, potentially altering the expression or activity of key enzymes in the glycolytic pathway, thereby affecting cellular utilization of glucose and energy metabolism. Research indicates that integrin β1 signaling activates the FAK–PI3K–Akt–mTOR signaling axis, promoting the overexpression of *Twist* in breast cancer cells and enhancing aerobic glycolysis [50]. Furthermore, integrin β2 enhances the glycolytic activity in cancer-associated fibroblasts (CAFs) through mechanical regulation of the PI3K/AKT/mTOR pathway, leading to increased extracellular lactate production [51]. Integrin β4 induces BNIP3L-dependent mitochondrial autophagy and lactate production in cancer-associated fibroblasts, thereby promoting breast cancer cell proliferation, epithelial–mesenchymal transition, and invasion [52]. The increase in cellular glycolysis induced by mechanical stress in hepatic sinusoidal endothelial cells, which can be attenuated by inhibiting integrin β1, further demonstrates the regulatory role of integrins in glycolysis [53]. The integrin β3–PKM2 pathway-mediated aerobic glycolysis has been reported to contribute to mechanical ventilation-induced pulmonary fibrosis [54]. Interestingly, the common aerobic glycolysis observed in tumors can lead to a decrease in extracellular pH, which in turn modulates integrin conformation and integrin-dependent cell adhesion and migration [55]. Therefore, the enhanced glycolysis mediated by integrins, as well as the glycolysis-regulated function of integrins, may form a positive feedback loop to promote the progression of pulmonary fibrosis.

YAP/TAZ act as sensors of cellular microenvironmental structure and mechanical characteristics, serving as important transcriptional regulators that are commonly activated in human malignancies. Their activation induces proliferation, drug resistance, and metastasis of cancer cells [56]. Although there is limited research on the relationship between YAP/TAZ and glycolysis, evidence suggests that they can promote the expression of key enzymes in the glycolytic pathway, thereby increasing glucose metabolism and elevating intracellular lactate production. This function has been widely reported [57,58]. The pharmacological and genetic inhibition of YAP leads to weakened glycolysis in cells, indicating the significant regulatory role of YAP in cellular glycolysis [59,60]. The activation of YAP/TAZ mediated by integrins/FAK signaling has been widely reported to regulate the progression of diseases [61,62,63]. Given that integrins/FAK and YAP/TAZ both regulate cellular glycolysis, the hypothesis that integrins/FAK regulate cellular glycolysis through the YAP/TAZ axis is reasonable. However, there is currently no definitive evidence to suggest that integrins reprogram cellular glycolysis through YAP/TAZ mediation to regulate disease progression. In fact, the activation of YAP/TAZ signaling has been shown to be dependent on glycolysis. When glucose metabolism is blocked or glycolysis is reduced, the transcriptional activity of YAP/TAZ is significantly decreased, and YAP/TAZ is essential for the full activation of glucose-promoted growth activity [64]. Interestingly, the activation of transcription factors YAP and TAZ (YAP/TAZ) mediated by TGF-β appears to be dependent on HK2, and the silencing of HK2 significantly attenuates the activation of TGF-β-induced YAP/TAZ signaling [44]. Therefore, the hypothesis that interferon regulates the transcriptional activity of YAP/TAZ through mediating enhanced glycolysis seems plausible, but it requires precise experimental data for support.

**Figure 2:**
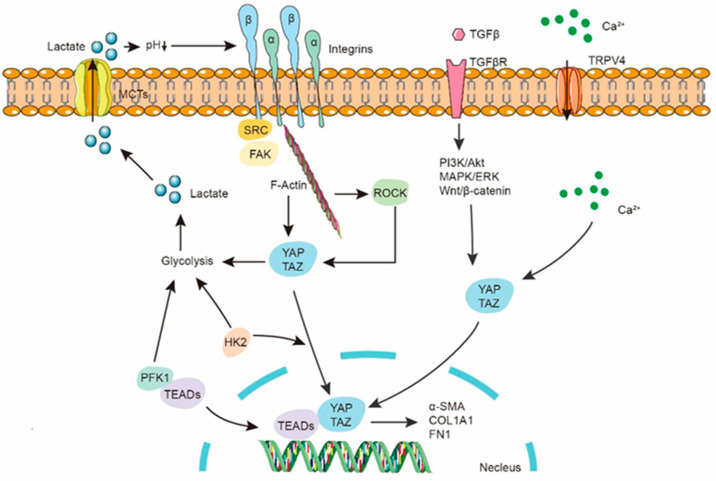
The role of YAP/TAZ in regulating glycolytic reprogramming in lung fibrosis. The integrin–FAK–YAP/TAZ signal axis promotes the expression of marker genes for lung fibrosis. YAP/TAZ promotes enhanced glycolysis in cells, and the enhanced glycolysis strengthens the nuclear translocation of YAP/TAZ. Additionally, the key glycolytic enzyme HK2 is necessary for the nuclear translocation of YAP/TAZ. PFK1 can bind to TEADs and promote their functional and biochemical cooperation with YAP/TAZ.

In addition, although it has been demonstrated that HK2 can accumulate in the nucleus of cancer cells, the exact function of nuclear HK2 remains incompletely understood [65]. Due to the similar metabolic reprogramming observed in myofibroblasts and cancer cells, it is also possible that HK2 may accumulate in the nucleus of IPF cells and influence targets involved in promoting pulmonary fibrosis. In addition to HK2, the activation of various human and murine fibroblast cell lines, as well as primary human lung fibroblasts, is often accompanied by significant upregulation of glycolytic enzymes such as PFK1 and PFKFB3. In fact, researchers have discovered that the enzyme phosphofructokinase-1 (PFK1) can interact with the YAP/TAZ transcriptional co-activator TEADs, thereby promoting their functional and biochemical cooperation with YAP/TAZ. This interaction may represent one of the pathways through which PFK1 regulates fibroblast differentiation [64]. The evidence above suggests that YAP/TAZ may play a crucial role in regulating the progression of pulmonary fibrosis through glycolytic reprogramming (Figure 2).

It is noteworthy that PFKFB3 is upregulated in fibrotic lungs, particularly in myofibroblasts of IPF patients. Additionally, significant increases in PFKFB3 expression have been detected in the lungs of mice treated with bleomycin or adenovirus. The enhanced glycolysis mediated by PFKFB3 leads to an increase in the intermediate metabolite succinate in the tricarboxylic acid cycle, stabilizing hypoxia-inducible factor 1-alpha (HIF-1α) and directly promoting myofibroblast differentiation [66]. Pharmacological inhibition of PFKFB3 not only suppresses fibroblast activation induced by TNF-α and TGF-β, but also alleviates pulmonary fibrosis in mice [66,67]. It is worth mentioning that similar to YAP/TAZ, HIF-1α also regulates *HK2* expression [68]. Further investigation into the relationship between HIF-1α and glycolytic enzymes can provide more insight into how glycolysis promotes the development of pulmonary fibrosis and expand therapeutic options. In summary, in both IPF and experimental pulmonary fibrosis, there is increased glycolytic activity and enhanced expression of glycolytic enzymes. Inhibiting the progression of pulmonary fibrosis by targeting these enzymes through genetic or pharmacological means appears to be a viable therapeutic strategy.

## 4. Lactate Production and Oxidation

Lactate is a classical byproduct of glucose metabolism, and its production primarily depends on glycolysis. Glucose in the cytoplasm undergoes a series of catalytic reactions to be converted into pyruvate. Pyruvate can either enter the mitochondria and participate in the TCA cycle or be converted to lactic acid in the cytoplasm by the action of lactate dehydrogenase. Lactic acid is considered a marker of increased glycolytic flux as it is an inevitable product of glycolysis. In addition to glycolysis, lactate can also originate from the metabolism of glutamine. Glutamine is first converted to glutamate, which is then acted upon by glutamate dehydrogenase to generate α-ketoglutarate (α-KG) for entry into the tricarboxylic acid (TCA) cycle. α-KG is subsequently converted to oxaloacetate, which is further converted to malate and exits the mitochondria. In the cytoplasm, malate is converted to NADPH and pyruvate by cytosolic malic enzyme (ME1). Finally, pyruvate is catalyzed to lactate [69,70]. Lactate is one of the most abundant byproducts of cellular metabolism in diseased tissues. For a long time, lactate was mistakenly believed to be a result of skeletal muscle hypoxia during contraction. It was not until the concept of aerobic glycolysis was introduced that the production of lactate under aerobic conditions was recognized. The concept of aerobic glycolysis also led to the understanding that the rapid production of lactate is considered a phenotype associated with cancer [71]. In fact, lactate production occurs not only in hypoxic muscles or rapidly proliferating cancer tissues, but also in most cultured mammalian cells, even under conditions of abundant oxygen, where glucose metabolism generates a significant amount of lactate [72,73]. In clinical diagnosis, lactate has been established as a diagnostic biomarker for various metabolic disorders. The elevation of lactate concentration in the human microenvironment often indicates impaired mitochondrial function and disruption of aerobic respiratory chain transmission. However, it is premature to consider lactate production as a direct result of mitochondrial dysfunction. The presence of aerobic glycolysis is more like a survival mechanism rather than an adaptive response [74]. In fact, the majority of lactate is utilized as a substrate to provide fuel for mitochondrial respiration in different cell types throughout the body [75,76]. The cellular consumption of lactate for ATP generation is achieved through the enzymatic activity of lactate dehydrogenase (LDH), which converts lactate into pyruvate. However, due to the reversible reaction mediated by LDH, cells ultimately achieve irreversible removal of lactate through the action of pyruvate dehydrogenase (PDH). PDH catalyzes the conversion of pyruvate into acetyl-CoA, which can then enter the tricarboxylic acid (TCA) cycle [77,78].

Interestingly, a substantial body of research has demonstrated that in healthy humans, several metabolically active organs prioritize lactate as a primary biological fuel [79,80,81]. The same is true in the lungs; even under normal physiological conditions, the lactate levels in the lungs are relatively higher compared to many other tissues. Additionally, through carbon labeling, it has been observed that under normal oxygen conditions, approximately 40% of all glucose consumed by the lungs in healthy rats is ultimately converted into lactate [82,83]. It has been proposed that lactate production can serve as an energy source for lung cells, especially for those lacking sufficient nutrition in the pulmonary circulation. Research has shown that normal AEC2s exhibit high oxidative capacity and can preferentially utilize lactate as a metabolic substrate for ATP production in mitochondria. AEC2s cultured in lactate have been found to have twice the cell growth rate compared with those cultured in glucose [84,85]. Furthermore, in lung and pancreatic tumors, lactate contributes more to the tricarboxylic acid cycle (TCA cycle) than glucose [86]. According to reports, in tumors surrounding the stromal fibroblasts that nourish tumor cells, there is an increase in lactate metabolism and production, and increased aerobic glycolysis in stromal cells leads to increased lactate production and secretion. Subsequently, lactate is transported to adjacent cancer cells through lactate transporters (also known as monocarboxylate transporters) and serves as a primary source of energy [86,87].

The development of pulmonary fibrosis can lead to respiratory distress in patients and impair gas exchange in the alveoli, which mistakenly suggests that the accumulation of lactate in fibrotic lungs is due to pulmonary hypoxia. However, studies by Routsi et al. indicate that the production of lactate in patients with acute lung injury is directly proportional to the severity of lung injury, but lactate production does not seem to be solely attributed to tissue hypoxia in the lungs [88,89]. Although hypoxia-inducible factors are significantly induced in IPF, the role of HIF proteins is not limited to hypoxic conditions. In the complex microenvironment of lung tissue, the sources of lactate are likely to be diverse, and its functions are diverse as well. Based on current theories, under normal conditions, when lung tissue is injured, AEC2s differentiate into AEC1s to promote re-epithelialization. Additionally, a large number of fibroblasts are recruited to secrete extracellular matrix components to facilitate wound healing. The main cells involved in these processes are expected to have enhanced metabolic capabilities. The repair of lung injury requires significant energy support for the highly active proliferation activities of both epithelial cells and fibroblasts. As previously reported, excessive production of lactate often occurs in cell populations with enhanced proliferative activity. Therefore, the elevated lactate levels in IPF lungs may be a consequence of sustained ineffective re-epithelialization and continuous activation of fibroblasts, rather than the cause of IPF. However, the abnormal accumulation of lactate in the lungs is undoubtedly detrimental to the organ, and the regulatory role of lactate in the process of pulmonary fibrosis remains complex and significant. In fact, researchers have reported numerous small molecule inhibitors targeting glycolysis or lactate, and have achieved promising results in experimental pulmonary fibrosis (Table 1). In the next section, we discuss the regulatory role of lactate in the major cell populations in the lungs and provide an overview of emerging evidence suggesting that targeting specific lactate metabolic pathways could be used for the treatment of IPF.

## 5. Lactate in Fibroblast

**Figure 3:**
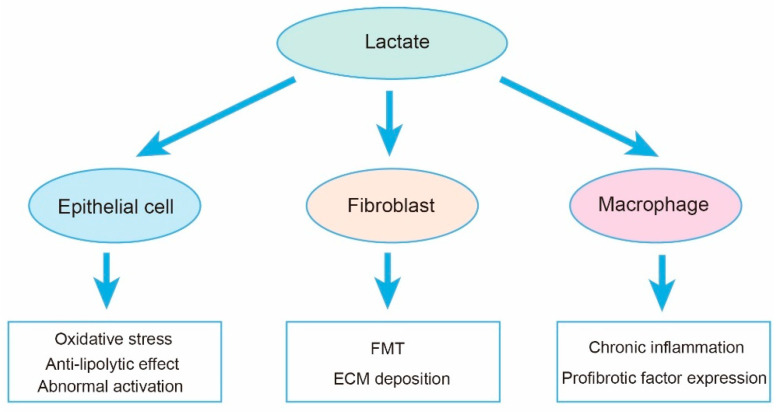
Lactate potentially regulates three major cell populations in the lungs, including the modulation of energy metabolism in epithelial cells, induction of fibroblast differentiation, promotion of macrophage polarization towards a pro-inflammatory phenotype, and regulation of histone acetylation. These regulatory effects contribute to the progression of IPF.

Studies have shown that lactate levels are increased in IPF lung tissue and in mouse models of bleomycin-induced fibrosis compared with healthy tissue. Many rate-limiting glycolytic enzymes are upregulated in fibroblasts of fibrotic lungs, promoting an increase in glycolytic flux, and the accumulation of lactate may play a significant regulatory role in the lungs, including its effects on various cell types (Figure 3). Specifically, myofibroblasts in fibrotic lungs exhibit enhanced glycolysis and cellular acidification [66,97,98,99]. Adding lactate to the culture medium of fibroblasts in vitro can activate TGF-β1, thereby promoting the differentiation of fibroblasts into myofibroblasts [100]. Although extracellular acidification and enhanced lactate production have been widely reported as key features of TGF-β1-induced activation of fibroblasts and are crucial for myofibroblast differentiation and collagen synthesis, they do not fully elucidate the sources and regulatory mechanisms of lactate production in the lungs.

Pyruvate is converted to lactate by the action of lactate dehydrogenase, with LDHA in particular favoring the production of lactate. In addition to the concentration of LDH protein itself, there are several factors that determine whether lactate is produced or consumed, and one of these factors is the expression of LDH isoforms. Specifically, LDH5, composed of four LDHA subunits, strongly supports the reduction of pyruvate to produce lactate [101,102]. In studies investigating the pH-dependent activation of TGF-β by lactate, it has been found that exogenously added TGF-β1 can induce the expression of LDH5 through HIF-1α, further promoting lactate production and differentiation into myofibroblasts in fibroblasts. Genetic inhibition of LDH5 or LDHA significantly blocks TGF-β-induced myofibroblast differentiation [100]. HIF-1α is a classical transcription factor that regulates glycolysis and has been shown to play a key role in the progression of fibrosis in various organs, including the lungs [103,104,105]. It has been reported that HIF-1α can activate PDK1, leading to increased phosphorylation of PDH and increased lactate production, thereby inducing myofibroblast differentiation, and specific knockout of HIF-1α in fibroblasts can alleviate bleomycin-induced pulmonary fibrosis in mice. Pharmacological inhibition of PDK1 restores PDH levels and reduces TGF-β-induced myofibroblast differentiation [92]. Additionally, treatment with the lactate dehydrogenase inhibitor gossypol attenuated the increased synthesis of α-SMA protein induced by TGF-β1 in control and fibrotic lung fibroblasts [94]. Gossypol not only inhibits fibroblast differentiation in vitro but also prevented the progression of experimental pulmonary fibrosis induced by bleomycin or radiation in mice [95,96]. Although gossypol has been used in studies to inhibit pulmonary fibrosis in mice, its non-specific cytotoxic and genotoxic effects in mammalian cells prevent its current clinical application [106,107]. Due to increasing evidence suggesting that metabolic reprogramming and elevated lactate levels may play a significant role in the pathogenesis of IPF, pharmacological inhibition of LDHA and LDH5 has emerged as a potential strategy to inhibit myofibroblast differentiation in IPF [107,108]. However, a subsequent study demonstrated that treatment of lung fibroblasts with a specific small molecule inhibitor of LDH5, compound 408 (Genentech, South San Francisco, CA, USA), effectively suppressed LDH5 activity and reduced aerobic glycolysis and lactate production. Nevertheless, inhibition of LDH5 did not decrease the production of fibronectin, collagen, and αSMA in primary human lung fibroblasts mediated by TGF-β1 [109]. Therefore, although there is dysregulation of glycolysis in IPF fibroblasts, it is likely that glycolytic dysfunction is not the sole driving factor of fibroblast-to-myofibroblast transition (FMT). The direct link between glycolytic regulation and TGF-β1-mediated FMT remains to be determined. These results suggest that inhibiting the aberrant glycolysis in fibroblasts may alleviate the progression of pulmonary fibrosis. In addition to focusing on glycolytic changes in fibroblasts, further exploration of metabolic dysregulation in various cell populations within the IPF microenvironment is warranted to develop more promising therapeutic strategies.

## 6. Lactate in AEC2s

Fibroblasts have been a focal point in IPF progression research, and enhanced glycolysis was initially detected in IPF fibroblasts. However, AEC2s, as a central factor in the development of pulmonary fibrosis, exhibit mitochondrial metabolic dysfunction, which is one of the characteristic features of IPF. Therefore, AEC2s may be one of the primary sources of lactate in the lungs of IPF patients. AEC2s from IPF lungs exhibit reduced activity of electron transport chain (ETC) complexes I and IV, leading to insufficient oxidative phosphorylation, decreased ATP production, and increased mitochondrial ROS generation [110,111]. In IPF lungs, AEC2s exhibit mitochondrial swelling and enlargement, along with reduced mitochondrial autophagy and biogenesis. Specifically, the increase in mitochondrial ROS (mtROS) leads to damage to mitochondrial DNA [112,113]. One of the main sources of mtROS production may be lactate. In pathological conditions, lactate, as a major mitochondrial energy substrate and electron donor, can increase mitochondrial ROS production through enhanced electron transport chain (ETC) activity during short-term cellular respiration [114]. Moreover, ROS generated from glycolysis can activate NLRP3 inflammasomes in lung epithelial cells, promoting the progression of radiation-induced lung injury [115]. Therefore, the dysregulation of lactate metabolism in AEC2 cells may be one of the key factors in pulmonary fibrosis, and it has been reported that alterations in lactate metabolism may be a potential characteristic of AEC2 dysfunction in IPF. Newton et al. examined the oxidative and glycolytic functions in primary epithelial cells isolated from IPF and control subjects and found that IPF AEC2s exhibited a relatively higher PPR/OCR ratio, indicating enhanced glycolysis in the lungs of IPF patients [116]. Similarly, the LDH isoenzymes LDH4 and LDH5, which are primarily responsible for lactate production, account for over 60% of the total LDH content in IPF AEC2s. Similar to fibroblasts, IPF-derived AEC2s exhibit increased oxygen consumption when LDHA is inhibited [116]. According to reports, in cystic fibrosis, mutations in CFTR reduce the activity of mitochondrial complex I and promote LDH activity, LDH expression, and increased lactate production in cells, and the increased lactate secretion appears to be a key factor contributing to extracellular pH reduction in the lungs, while inhibition of LDHA restores extracellular pH levels [117,118].

Although lactate produced by glycolysis may be one of the main sources of ROS in cells, its presence helps maintain redox homeostasis and the integrity of tissues and the entire organism. Generally, the ratio of lactate to pyruvate in the cell is used as an indicator of the cytoplasmic ratio of NADH to NAD, which exist in oxidized and reduced forms in the cell [119]. NAD is an essential coenzyme in major energy metabolism processes such as oxidative phosphorylation, fatty acid β-oxidation, and the tricarboxylic acid cycle. It has been reported that a decrease in the NAD/NADH ratio may lead to cellular senescence in AEC2s and promote pulmonary fibrosis [120]. An increase in the NAD/NADH ratio has been shown to inhibit bleomycin-induced inflammation and fibrotic responses in the lungs of mice [121]. Lactate, as a metabolic intermediate, is generated and eliminated through a specific process involving the oxidation of NADH to NAD+ and H+, which helps maintain electron flux. This process is accompanied by the catalytic action of LDH in converting lactate to pyruvate [119,122,123,124]. When intracellular lactate increases, it may lead to an increase in the NAD/NADH ratio. In this situation, through the transport and oxidation of lactate, cells can utilize lactate as an energy source and reoxidize NADH to NAD+, thereby maintaining cellular energy metabolism and redox balance [125]. Moreover, lactate supplementation through the action of lactate dehydrogenase replenishes cellular NAD+ levels, stimulating the continued progression of glycolysis to generate ATP at a faster rate [124,126]. In addition, LDH and MCT help maintain a consistent cytoplasmic NAD to NADH ratio in each cell, which is in line with the overall pyruvate to lactate ratio, effectively buffering the levels of NAD and NADH in each cell [127]. In conclusion, lactate plays a crucial role in maintaining the balance between NAD and NADH, ensuring that cells can adapt to different metabolic states and maintain normal energy metabolism and redox status. However, the metabolic abnormalities in IPF alveolar epithelial cells may be one of the reasons for the elevated lactate levels in the lungs. Furthermore, while lactate is normally utilized as fuel by healthy alveolar epithelial cells to generate the required energy for cellular activities, the dysregulated lactate metabolism in AEC2 cells may be a primary driving factor in IPF. Manipulation of lactate metabolism could potentially serve as an intervention strategy to reverse this debilitating disease.

## 7. Lactate in Inflammatory Cells

Typically, lactate accumulates in chronic inflammatory sites, particularly in inflamed joints, atherosclerotic plaques, and multiple sclerosis, where elevated lactate levels have been detected [128,129,130,131]. Historically, IPF was considered a chronic inflammatory disease. However, subsequent research has shown that purely anti-inflammatory treatments, such as steroids, do not improve IPF and may even have harmful effects on patients in clinical trials [12,132,133]. However, all stages of fibrosis are accompanied by innate and adaptive immune responses, and the observed inflammatory changes in IPF may occur independently of the primary fibrotic remodeling process. Current treatment approaches, such as pirfenidone and nintedanib, can also exert their effects by modulating the inflammatory processes. It is important to maintain an appropriate balance between inflammation and its complete resolution, as impaired resolution may lead to a chronic inflammatory state, which can subsequently result in fibrosis [134,135]. It can be affirmed that inflammatory cells are active in fibrotic lungs, and inflammation is typically necessary for the resolution of lung injury, such as infection or physical trauma. Therefore, inflammatory cells may be one of the sources of lactate in the lungs. Investigations of sputum and bronchoalveolar lavage fluid from patients with cystic fibrosis may reveal potential sources of lactate. During chronic inflammatory stimulation, activated neutrophils undergo necrotic decay, leading to the release of various components from neutrophil granules and cytoplasmic solutes, including a significant amount of lactate in airway secretions [136]. Dieter detected lactate concentrations in sputum similar to those reported by Kottman, and lactate was also detected in BALF. These lactate levels were found to be correlated with neutrophil influx and concentrations of myeloperoxidase and elastase [137]. During the process of pulmonary fibrosis, lung macrophages commonly exhibit enhanced glycolysis and a high level of lactate production, and alterations in glycolysis in the lungs may regulate the activation and function of macrophages in type 2 immunity [138]. As previously reported, glycolysis is upregulated in macrophages of fibrotic lung tissue, characterized by increased expression of various glycolytic enzymes in fibrotic lung macrophages. Additionally, these macrophages exhibit M2-like characteristics, which are known to promote fibrosis [40], and enhanced glycolysis promotes inflammasome activation, leading to acute exacerbation of bacterial infection-induced pulmonary fibrosis [39]. Additionally, increased glycolysis can promote macrophage polarization, thereby contributing to acute lung injury in rats [139]. Based on current understanding in the field of biomedical science, it appears that lactate induces sustained chronic inflammation in the lungs and regulates abnormal activation of macrophages. Inhibiting macrophage glycolysis may be a potential approach to suppress early-stage inflammation in pulmonary fibrosis.

## 8. Lactate and Epigenetics

Lactate is not only an active metabolic byproduct but has also been reported as a precursor molecule for post-translational protein modifications. Zhao first reported lysine lactylation (Kla) as a modification of histones in macrophages, playing a role in gene transcription regulation [140]. Histone lactylation modification plays a crucial role in regulating various cellular processes, especially mediating the reprogramming of immune cells. Histone lactylation has been shown to increase in cells stimulated by interferon-gamma (IFN-γ), lipopolysaccharide (LPS), or bacterial stimuli, promoting the expression of genes associated with tissue repair, such as arginase-1 (*Arg1*) and *KLF4* [141,142,143]. It has been reported that defects in lactate production may lead to a decrease in histone lactylation, thereby affecting the transcriptional regulatory capacity of macrophages and reducing the expression of genes related to tissue repair, ultimately weakening the reparative transformation ability of macrophages [144]. In lactate-treated Th17 cells, there is an elevation in histone H3K18 lactylation levels. This lactylation modification has the ability to reprogram the pro-inflammatory T-cell phenotype into a regulatory T-cell phenotype, thereby suppressing the pathogenicity of Th17 cells during inflammation [145]. In the late stage of M2 macrophage polarization, there is an increase in histone lactylation (Kla) in the promoter regions of relevant genes. This promotes the transition of macrophages from an inflammatory phenotype to a homeostatic phenotype [146,147]. Additionally, lactate has the ability to increase H3K18 lactylation in macrophages, thereby activating the transcription of target genes and leading to M1 macrophage polarization and inhibition of the NLRP3 inflammasome [148,149]. Furthermore, lactate can inhibit the Warburg effect by activating lactylation of PKM2, thereby inducing a transition of pro-inflammatory macrophages towards a reparative phenotype [150]. These findings seem contradictory to previous reports suggesting that lactate is a key molecule promoting the progression of chronic inflammation, and lactate appears to inhibit the development of inflammation and promote tissue repair. However, these results also indicate that lactate exhibits diverse functions in different tissues or microenvironments. This diversity of functions may be related to whether lactate levels are within a tolerable range for a given tissue. High levels of lactate can induce SNAIL1 lactylation, promoting EndoMT (endothelial-to-mesenchymal transition), thereby enhancing cardiac fibrosis and exacerbating cardiac dysfunction [151]. Elevated levels of lactate in the circulation stimulate the lactylation and acetylation of HMGB1, leading to its release from macrophages through exosome secretion. This further disrupts endothelial integrity and increases vascular permeability [152].

Aberrant elevation of lactate-induced histone lactylation plays a crucial role in the progression of IPF. Increased lactate levels in human alveolar macrophages lead to an increase in Kla. Moreover, in a mouse model of bleomycin-induced pulmonary fibrosis, the elevated lactate levels in BALF induced histone lactylation in the promoter regions of pro-fibrotic genes in macrophages, thereby promoting the development of pulmonary fibrosis [153]. Similarly, in nitrite-related IPF (As-IPF), high levels of lactate increase the overall lactylation level in fibroblasts, promoting their transformation into myofibroblasts. In alveolar epithelial cells (AECs), lactylation of H3K18 regulates the transcription of target gene H3K18la, which enhances the secretion of TGF-β1 and promotes the transformation of fibroblasts into myofibroblasts [154]. Although there have been numerous studies on histone lactylation, the detailed regulatory mechanisms between lactylation and pulmonary fibrosis remain poorly understood. Kla may regulate the expression of pro-fibrotic mediators during the process of pulmonary fibrosis. In-depth investigation of the relationship between protein lactylation and pulmonary fibrosis will contribute to a better understanding of the disease’s pathogenesis and provide new insights for future therapeutic approaches.

## 9. Lactate Shuttle and Multicellular Crosstalk in Fibrotic Lungs

Based on the hypothesis proposed by Brooks, lactate produced by organs in the body can be transported in the circulation, and lactate signaling can be transduced between different cells, tissues, and organs. This phenomenon is known as the lactate shuttle, which involves the exchange and utilization of lactate as a metabolic fuel and signaling molecule. The lactate shuttle plays a crucial role in coordinating energy metabolism and cellular communication throughout the body [155,156,157,158]. IPF is considered to be the result of multiple cell interactions. Various cell types, including inflammatory cells, fibroblasts, epithelial cells, and immune cells, are involved in the development of pulmonary fibrosis. These cells interact with each other through cell signaling and the release of cytokines, collectively regulating the progression of pulmonary fibrosis. Therefore, the lactate shuttle may play a crucial role in the development of IPF. Studies have found that lactate accumulates in the lung tissue of patients with pulmonary fibrosis and is closely associated with pathological processes such as inflammation, fibrosis, and cell apoptosis. Additionally, the lactate shuttle is involved in the interaction between cell types associated with pulmonary fibrosis, such as pulmonary fibroblasts and inflammatory cells. Lactate affects biological processes such as proliferation, migration, and differentiation of cells involved in pulmonary fibrosis by regulating intracellular signaling pathways and gene expression. Cui et al. reported that lactate derived from fibrotic lung myofibroblasts plays a crucial role in promoting the pathological phenotype of macrophages in the lung. Lactate derived from myofibroblasts induces p300-mediated histone lactylation and upregulates fibrosis-related gene expression in macrophages [153]. Lactate is primarily produced in the cytoplasm during aerobic glycolysis, especially in hypoxic conditions or proliferating cells. It is then transported across the plasma membrane through lactate transporters, mainly dependent on the regulation of monocarboxylate transporters (MCTs) such as MCT1-4 [159,160]. The lactate transporter protein family consists of multiple subtypes, with the most important ones being monocarboxylate transporter 1 (MCT1) and monocarboxylate transporter 4 (MCT4). MCT1 is primarily found in tissues such as the heart, liver, and kidneys, while MCT4 is predominantly present in muscle and nerve tissues. These proteins regulate the transport of lactate, maintaining the balance of lactate concentration inside and outside the cells [161,162]. Particularly in human cancer tissues, MCT1 and MCT4 are highly expressed, which may be associated with maintaining the acidic pH of the tumor microenvironment [163,164,165]. Generally, MCT subtypes 1-4 are involved in the bidirectional transport of lactate and pyruvate, but different subtypes have different preferences. MCT1 is primarily responsible for mediating the uptake of lactate into cells, while MCT4 tends to facilitate the efflux of lactate from cells [166,167,168]. Under physiological conditions, the synergistic action of MCT1-4 facilitates the shuttling of lactate between glycolytic cells and oxidative cells [169]. This is a key factor in maintaining lactate homeostasis in different tissues. MCT1 and MCT4 have both been shown to be present in adult whole lung samples [170], and type II alveolar epithelial cells have been demonstrated to express them. This may be related to their preference for utilizing lactate as an energy source. Inhibition of MCT leads to a decrease in oxygen consumption in lactate-cultured cells, indicating the importance of MCT in lactate oxidation [84,171]. Although there is currently a lack of research on the MCT family in IPF, abnormal expression or inactivation of MCT can lead to abnormal insulin secretion, disruption of blood glucose regulation, and lactate transport defects [172,173]. Moreover, the high expression of MCTs is closely associated with cancer development. Targeted inhibition of the MCT family has become a viable cancer therapy approach. By inhibiting the MCT family, intracellular pH can be restored, leading to the suppression of cancer cell invasion and induction of tumor cell death [174,175,176]. In summary, the functions of lactate transporters extend beyond lactate transport alone, as they also play a role in establishing intracellular connections. Intracellular connections are crucial pathways for intercellular communication and substance exchange, influencing the stability and functionality of these connections.

**Figure 4:**
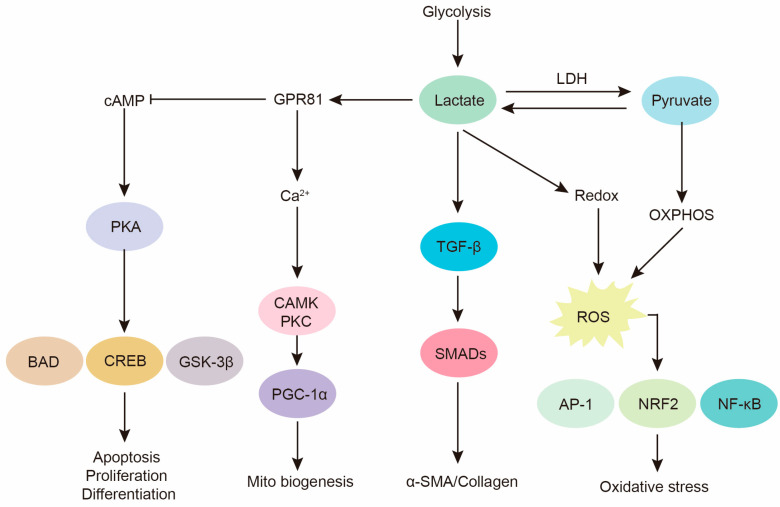
Diagram illustrating the impact of lactate on intracellular signal transduction. Lactate mediates pH-dependent activation of TGF-β, thereby promoting the expression of fibrosis mediators mediated by SMADs proteins. The accumulation of lactate and the progression of oxidative phosphorylation leads to the generation of ROS, thereby inducing the activation of oxidative stress signaling pathways. Lactate can increase intracellular Ca^2+^ through GPR81 mediation, activate calcineurin, further enhancing CaMK activity, and induce mitochondrial biogenesis. Additionally, the activation of GPR81 inhibits intracellular cAMP levels, leading to decreased PKA activity.

In addition to its transport through MCTs, lactate has been reported to act as a signaling molecule with autocrine, paracrine, and endocrine-like effects, earning it the name “lactormone” [177,178]. Research has shown that lactate plays a crucial regulatory and signaling role within cells, participating in multiple signal transduction processes, thereby influencing cellular metabolism, proliferation, and apoptosis, among other physiological processes (Figure 4). Furthermore, the lactate receptor GPR81, now known as hydroxycarboxylic acid receptor 1 (HCAR-1), is capable of regulating lactate signal transduction and mediating various biological processes such as lactate-induced energy metabolism, lipid accumulation, neuronal protection, and immune regulation [179,180,181,182,183]. HCAR1 is a G protein-coupled receptor that is expressed on the cell membrane and can bind to lactate, triggering intracellular signal transduction pathways. Lactate, through the activation of HCAR1, can promote tumor cell proliferation, invasion, and metastasis, while inhibiting immune cell function, thereby providing favorable conditions for tumor growth and metastasis [184,185,186]. Additionally, the lactate-GPR81 signaling pathway also plays an important role in pulmonary fibrosis. It has been reported that hypoxia induces the expression of GPR81 in lung fibroblasts, and lactate can act as a paracrine intercellular signal in the hypoxic microenvironment, independently of pH, to promote the differentiation of normal fibroblasts into myofibroblasts through GPR81 [187]. Fibrosis-associated mesenchymal progenitor cells (MPCs) express lactate receptor GPR81. Hypoxia-induced lactate production enhances the expression of GPR81 in IPF MPCs and promotes fibrotic processes mediated by MPCs in vivo. In contrast, inhibition of GPR81 significantly suppresses pulmonary fibrosis in mice [188].

GPR81 is primarily expressed in adipose tissue and other tissues and is well known for its anti-lipolytic effects. When lactate binds to GPR81, it can inhibit the lipolysis process in adipocytes through G protein-mediated signaling [189,190]. Interestingly, in IPF lungs, elevated lactate levels are accompanied by dysregulation of lipid metabolism, and the accumulation of lipids in the lungs promotes the progression of IPF [191,192,193]. This easily leads to the hypothesis that the elevation of lactate in IPF may contribute to the dysregulation of lipid metabolism in the lungs through its binding to GPR81. Reports suggesting lactate as one of the main sources of lipids have gained significant attention in the field [193,194]. In fact, early studies have already identified lactate as a potential substrate for pulmonary lipids, which has sparked further research into the role of lactate in the lungs and its relationship with lipid metabolism [85]. The discovery of lactate as a potential substrate for pulmonary lipids in the lungs provides a new perspective for understanding the regulatory mechanisms of pulmonary lipid metabolism. Further research will help uncover the interaction between lactate and GPR81 in the lungs, as well as their impact on lipid metabolism. This will contribute to a better understanding of the mechanisms underlying lipid metabolism dysregulation in pulmonary diseases and provide new targets and strategies for their treatment.

## 10. Role of Lactate in Tissue Repair and Regeneration

For a long time, lactate has been considered a villainous character in clinical and basic research due to misunderstandings about lactate metabolism. In both clinical and basic research, lactate has been regarded as a marker of cellular metabolic dysfunction and disease progression. However, as research has progressed, it has been gradually recognized that lactate metabolism and signaling may play a positive role in tissue repair and patient prognosis. Particularly in the treatment of sepsis, lactate can support blood pressure and circulation, aid in the delivery of antibiotics and energy substrates (gluconeogenesis precursors), and exhibit anti-inflammatory effects [195,196]. Additionally, as mentioned earlier, lactate has the ability to inhibit the development of inflammation and promote the transition of macrophages from a pro-inflammatory phenotype to a reparative phenotype. Furthermore, lactate has a positive effect on tissue regeneration in various damaged tissues, as it stimulates the promotion of VEGF-related protein levels and angiogenesis in endothelial cells [197,198]. Lactate also has the ability to activate muscle satellite cells to stimulate muscle hypertrophy and promote skeletal muscle regeneration in mice [199]. Pulmonary fibrosis is believed to be the result of impaired lung tissue regeneration. We speculate that during this process, an appropriate level of lactate may have a positive impact on lung tissue repair through mechanisms such as promoting angiogenesis, regulating immune responses, and exerting anti-inflammatory effects. However, abnormal accumulation of lactate may interfere with this repair process, leading to incomplete regeneration. Although the beneficial effects of lactate metabolism and signaling are gradually being recognized and understood, further research is still needed to uncover the detailed mechanisms and regulatory networks involved.

## 11. Conclusions

Cellular metabolic dysregulation has been shown to be involved in various pathological processes, but research on metabolic dysregulation associated with organ fibrosis, including IPF, is still lacking. In the lung tissue of IPF patients, the glycolytic pathway is reprogrammed, leading to a shift in cellular metabolism from oxidative phosphorylation to glycolysis. This allows cells to generate lactate as an energy substrate to meet their energy demands. However, abnormal activation of glycolysis may impact the physiological state of major cell populations in the lung, including through metabolic regulation, signal transduction, and inflammation modulation, thereby contributing to the development of idiopathic pulmonary fibrosis. Additionally, the abnormal activation of the glycolytic pathway can result in the accumulation of lactate in the lungs, and elevated levels of lactate in the lung lead to an increase in pro-fibrotic events.

**Figure 5:**
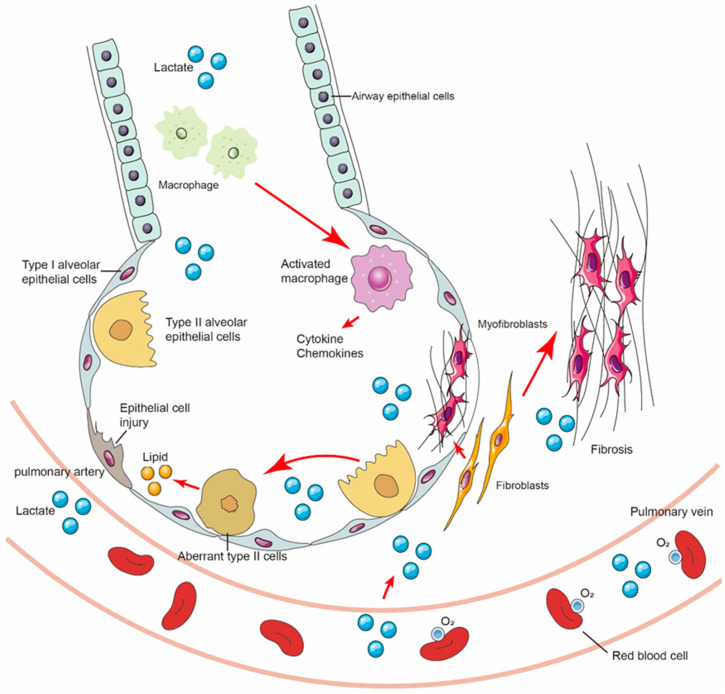
Lactate shuttling plays a role in the regulation of physiological and pathological processes in the lungs. In conditions of lung injury, elevated glycolysis in the lungs leads to the production of lactate by various cell types, which enters the alveolar environment. This lactate induces polarization of macrophages, resulting in the production of pro-fibrotic factors, recruitment of interstitial cells, and activation of fibroblasts. Lactate stimulation also leads to abnormal proliferation or apoptosis of epithelial cells, causing disruption of pulmonary epithelial integrity. Furthermore, the elevated levels of lactate in the lungs result in insufficient breakdown of lipids within the alveoli, leading to the accumulation of a large amount of lipids in the alveolar space. Lactate from the systemic circulation enters the pulmonary vasculature and contributes to a decrease in pH in the lung environment, promoting lung fibrosis development.

In the past, it was widely believed that an increase in lactate concentration in the body indicated that tissue cells were in an anaerobic state. However, the understanding of lactate as a metabolically valuable carbohydrate has now replaced this traditional view. Lactate is an inevitable product of glycolysis and can also be transported as an energy substrate to other cells or tissues, providing energy for cellular metabolism. Lactate in the lungs can regulate cell function and signal transduction through its binding to the receptor GPR81. The effects of lactate may include promoting angiogenesis, regulating immune responses, and exerting anti-inflammatory effects, all of which have positive impacts on lung tissue repair and regeneration. The abnormal accumulation of lactate in the lungs may be closely associated with the development of pulmonary fibrosis. Lactate is considered to be a signaling molecule that promotes fibrosis, and its shuttling in the lungs is a key event in regulating the progression of pulmonary fibrosis (Figure 5). It can directly stimulate the proliferation of lung fibroblasts and the synthesis of collagen and other fibrotic molecules, thereby promoting fibrosis progression. Additionally, lactate can enhance the release of inflammatory factors and the extent of inflammation in alveolar macrophages, promoting early-stage inflammation in pulmonary fibrosis and further exacerbating fibrosis development. Additionally, lactate may inhibit lipolysis, decrease fatty acid oxidation, and promote lipid synthesis through its interaction with the receptor GPR81, and this can lead to abnormal lipid metabolism and accumulation in the lungs. This process may be a key factor in the development of IPF, but further research is needed to confirm this. Importantly, we can no longer simply consider lactate as a metabolic waste product. Lactate plays an important role in cellular metabolism and signal transduction, with critical implications for cellular function and tissue repair in the lungs and patient prognosis. Understanding the regulatory mechanisms of lactate in the lungs is of significant importance for the diagnosis and treatment of IPF. 

## Figures and Tables

**Table 1:** Glycolysis inhibitors demonstrate preclinical anti-fibrotic effects in IPF.

Target	Drug	Preclinical Data in IPF
PFKFB3	3PO	↓ The activation of fibroblasts,
		↓ lung fibrosis in mice [66,90].
HK2	Lonidamine	↓ TGF-β-induced fibrosis,
		↓ Fibrosis marker,
		↑ Lung function in mice [44].
	2-DG	↓ Lactate level,
		↓ collagen synthesis [90].
Glycolysis	KD025	↑ OXPHOS,
		↓ Lactate level,
		↓ pulmonary endothelial permeability [91].
GLUT1	GLUT inhibitor II	↓ PAI-1, CTGF, and α-SMA [37].
	Phloretin	↓ α-SMA, collagen, and fibronectin,
		↓ lung fibrosis in mice [37,38].
PDK1	Dichloroacetate	↓ Lactate and α-SMA production,
		↓ lung fibrosis in mice [92].
PKM2	Naphthoquinone derivatives	↓ TGF-β signal,
		↓ collagen deposition [93].
LDHA	Gossypol	Lactate production,
		fibroblast differentiation,
		pulmonary fibrosis in mice [94,95,96].

## Data Availability

The datasets presented in this study can be found in online repositories. The names of the repository/repositories and accession number(s) can be found in the article.
